# Remdesivir in Severe COVID-19 and Non-Invasive Ventilation: A Real-Life Experience

**DOI:** 10.3390/healthcare9091108

**Published:** 2021-08-27

**Authors:** Francesca Simioli, Carmine Nicoletta, Maria Rosaria Valentino, Maria Martino, Anna Annunziata, Novella Carannante, Pierpaolo Di Micco, Giuseppe Fiorentino

**Affiliations:** 1 Sub-Intensive Care Unit, Department of Respiratory Pathophysiology and Rehabilitation Monaldi–A.O. Dei Colli, 80131 Naples, Italy; francesimioli@gmail.com (F.S.); carmine.nicoletta@gmail.com (C.N.); mrvalentin@gmail.com (M.R.V.); mariamartino@gmail.com (M.M.); anna.annunziata@gmail.com (A.A.); giuseppefiorentino1@gmail.com (G.F.); 2 Emergency Unit, Department of Infectious Diseases, Cotugno Hospital, 80131 Naples, Italy; carannantenovella@gmail.com; 3 UOC Medicina, Buonconsiglio Fate Bene Fratelli Hospital of Naples, 80123 Naples, Italy

**Keywords:** antiviral, SARS-CoV2, ARDS, NIV, CPAP

## Abstract

Background: Antiviral treatment is a hot topic regarding therapy for COVID-19. Several antiviral drugs have been tested in the months since the pandemic began. Yet only Remdesivir obtained approval after first trials. The best time to administer Remdesivir is still a matter for discussion and this could also depend upon the severity of lung damage and the staging of the infection. Methods: We performed a real-life study of patients hospitalized forCOVID-19 and receiving non-invasive ventilation (NIV). In this single-center study, a 5 day course of Remdesivir was administered as compassionate use. Further therapeutic supports included antibiotics, low molecular weight heparin and steroids. Data collection included clinical signs and symptoms, gas exchange, laboratory markers of inflammation, and radiological findings. Major outcomes were de-escalation of oxygen-support requirements, clinical improvement defined by weaning from ventilation to oxygen therapy or discharge, and mortality. Adverse drug reactions were also recorded. All data were collected during hospitalization and during a 20-day follow up after treatment. Results: 51 patients were enrolled. A global clinical improvement was recorded in 22 patients (43%) at 12 days, and 36 (71%) at 20 days; in particular, at 12 days, 27 patients (53%) also had a de-escalation of oxygen-support class from a therapeutic point of view. Remdesivir use was associated with a lower hazard ratio for clinical improvement in the elderly (older than 70 years) and in subjects with more extensive lung involvement (total severity score at HRCT of more than 14). The 20-day mortality was 13%. Conclusions: Results demonstrated that Remdesivir is associated with an improvement in clinical, laboratory and radiological parameters in patients with severe COVID-19 and showed an overall mortality of 13%. We conclude that, in this cohort, Remdesivir was a beneficial add-on therapy for severe COVID-19, especially in adults with moderate lung involvement at HRCT.

## 1. Background

Since December 2019, a novel coronavirus named SARS-CoV2 spread worldwide leading to a pandemic. The consequent disease was named COVID-19. It is usually characterized by respiratory symptoms due to the strong tropism of the virus for epithelial cells of the respiratory tract [1]. According to data released by WHO on 12 July, COVID-19 has affected more than 186 million people and has caused 4 million deaths worldwide [2].

More severe presentation is commonly related to respiratory involvement. It was estimated that approximately 9.4% of COVID-19 patients show respiratory failure and Acute Respiratory Distress Syndrome (ARDS) [3]. Vital signs on admission, including SpO2 < 90%, respiratory rate > 20 breaths/min, and heart rate > 100 beats/min, were also associated with poor outcomes [4]. Furthermore, older patients with COVID-19 have been reported to exhibit relatively higher mortality and severity of illness than younger patients [5]. Concomitant conditions may play a major role in determining a critical illness. Hypertension, diabetes mellitus, Chronic Obstructive Pulmonary Disease (COPD) and obesity have been described as risk factors for severity in meta-analysis [6]. Liu et al. found that the viral load was crucial in determining the disease severity, especially strongly correlated with lung injury Murray score [7]. This observation highlights the importance of antiviral therapy in severe COVID-19. Furthermore, viral load may also have a prognostic role in COVID-19 because of its correlation not only with infectivity, but with morbidity and mortality [8]. Viral load at diagnosis has been identified as an independent predictor of mortality in a large hospitalized cohort [9]. So the idea of associating qualitative testing with quantitative measurement of viral load may help clinicians in risk-stratifying patients and evaluating the chance of beginning an antiviral therapy. Several trials on COVID-19 evaluated the potential advantage of treating these patients with antivirals, and Remdesivir is one of them [10]. Remdesivir is a nucleotide analogue pro-drug that inhibits viral RNA polymerases. Few studies are available on this drug in a clinical setting with critical COVID-19 and for this reason the eligibility for Remdesivir remains uncertain, especially in severe cases. At the same time severe illness is the stage that requires more therapies and leads to a worse prognosis. Therefore, the aim of this study is to report on the effectiveness and safety of Remdesivir in a real-life setting with ventilated subjects.

## 2. Patients and Methods

This is a single center retrospective study on severe COVID-19. We included 51 patients aged more than 18 years that received Remdesivir for compassionate use. Inclusion criteria were hospitalization, and SARS-CoV-2 infection confirmed by reverse-transcriptase–polymerase-chain-reaction assay, ARDS; all patients, in fact, received a supportive therapy consisting of non-invasive ventilation (NIV) such as continuous positive airway pressure (CPAP) or high flow nasal cannula (HFNC). In addition, patients were required to have a creatinine clearance above 30 Ml per minute and serum levels of alanine aminotransferase (ALT) and aspartate aminotransferase (AST) less than five times the upper limit of the normal range in order to escape alterations caused by pharmacokinetics and clearance of the drug. All patients provided informed consent for this study. All patients received a 5-day course in intravenous Remdesivir. It was administered according to the following protocol: loading dose of 200 mg on day 1 and 100 mg daily for the following 4 days. Besides antiviral treatment and NIV, all patients received a supportive therapy consisting of intravenous methylprednisolone, subcutaneous enoxaparin and azithromycin.

**Figure 1:**
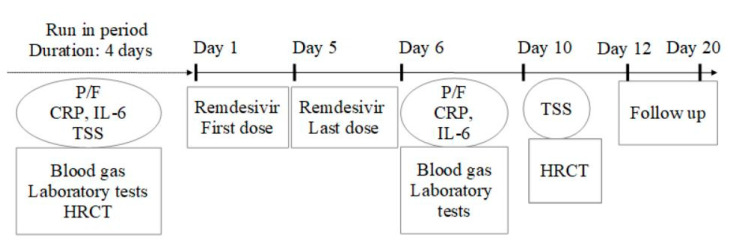
Study protocol of Remdesivir treatment initiation and follow up. P/F: pO2/FiO2 ratio. HRCT: high resolution computed tomography. CRP: C-reactive protein. IL-6: interleukin-6. TSS: total severity score.

Several markers of COVID-19 were selected as predictive of disease severity and included laboratory and radiological findings. The study assessments at baseline were performed within 4 days before Remdesivir initiation; it included PaO2/FiO2 ratio (P/F) obtained by blood gas analysis; C-reactive protein (CRP) and interleuchin-6 (IL-6) obtained by venous sampling. A radiologic assessment was also performed to define a baseline total severity score (TSS) by a high-resolution computed tomography (HRCT) of the chest. This was a visual quantitative evaluation based on summing up the extension of acute lung inflammatory lesions in each lobe, with a score of 0 (0%), 1 (1–25%), 2 (26–50%), 3 (51–75%), or 4 (76–100%), respectively. The TSS was reached by summing the five lobe scores. Blood gases and laboratory tests were repeated at day 6 (the day after the last administration of Remdesivir). The HRCT was performed again at day 10 (5 days after Remdesivir) (Figure 1).

The follow-up continued at least 15 days after the last dose of the treatment or until discharge or death. During this period, other data were collected such as changes in oxygen-support requirements (from NIV to room air or low-flow oxygen), hospital discharge, or death. Clinical improvement was defined as weaning from ventilation to oxygen therapy or discharge.

The study has been approved by the local ethics committee of University of Campania “Luigi Vanvitelli” and A.O. dei Colli in accordance with the 1976 Declaration of Helsinki and its later amendments.

Results are reported as number and percentage for categorical variables and median and interquartile range (IQR) for continuous variables and were collected for the following statistical analysis. Differences before and after the investigational drug in the full cohort were tested by the one-way ANOVA test. Statistical significance was tested by Tukey’s honestly significant difference test, with a *p*-value < 0.05. Clinical improvement in the Remdesivir compassionate-use cohort was described with the use of Kaplan–Meier analysis. Associations between baseline characteristics, such as age and radiologic extension of the disease, with clinical improvement were evaluated with a log rank test.

## 3. Results

**Figure 2:**
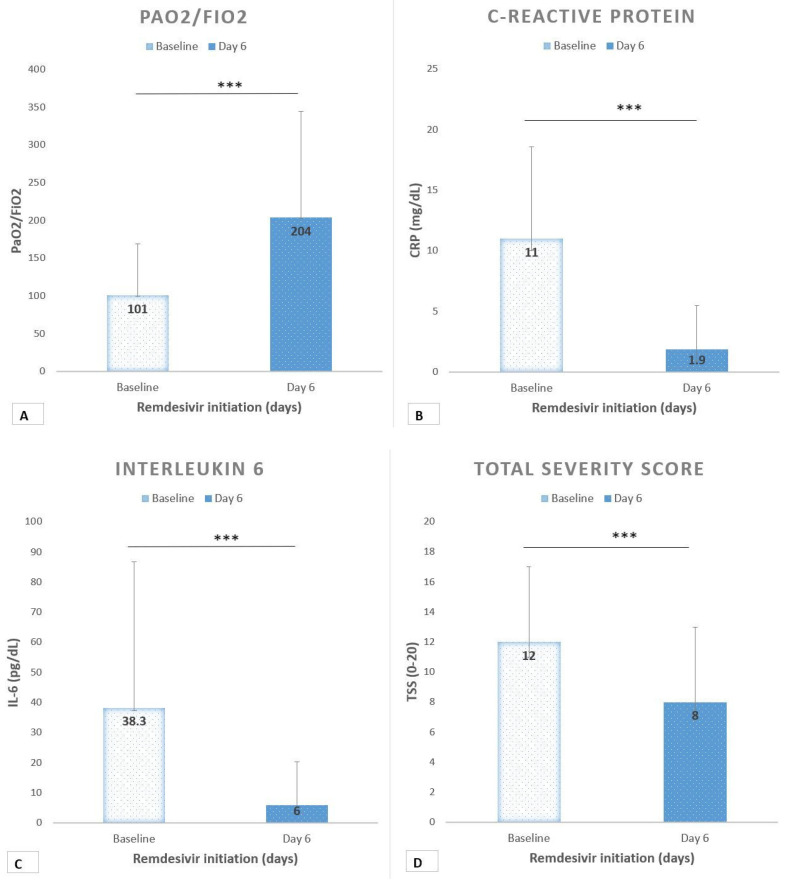
(**A**) Ratio of arterial oxygen partial pressure to fractional inspired oxygen (PaO2/FiO2) trend before and after Remdesivir. ***: *p* < 0.0001. (**B**) C-reactive protein before and after Remdesivir. ***: *p* < 0.0001. (**C**) Interleukin-6 before and after Remdesivir. ***: *p* < 0.0001. (**D**) Radiologic total severity score before and after remdesivir. ***: *p* < 0.0001.

Results are reported in Table 1 and included data regarding clinical characteristics at baseline, comorbidities, laboratory markers, oxygen support and radiological findings. The modification of several parameters is also reported in Figure 2. Fifty-one patients received a 5 day course of Remdesivir. Of these, 45 patients (88%) were men, the age range was 25 to 85 years, and the median age was 64 years (IQR 17). The median BMI was 28 (7). Eight patients had no comorbidity, 16 had one comorbidity, and 27 patients had two or more comorbidities. Baseline characteristics and the most common concomitant conditions are reported in Table 1. At baseline, the median P/F was 101 (68) and all patients were receiving NIV; in fact 40 (78%) were on CPAP and 11 (22%) on HFNC. The median duration of ventilation before the initiation of Remdesivir treatment was 3 days.

Baseline CRP was 11 mg/dL (7.6) and IL-6 was 38 pg/mL (48.5). After Remdesivir, the median P/F was 204 (141) with a mean increase of +103 (*p* < 0.0001) (Figure 2A). Inflammatory markers showed a decreasing trend. CRP after treatment was 1.9 mg/dL (3.6) with a mean change of −9 (*p* < 0.0001) (Figure 2B). IL-6 was 6 pg/dL (14.2) with a mean change of −32 (*p* < 0.0001) (Figure 2C). Moreover, the radiologic TSS significantly improved after treatment, with a median score of 8 (*p* < 0.0001) (Figure 2D).

**Figure 3:**
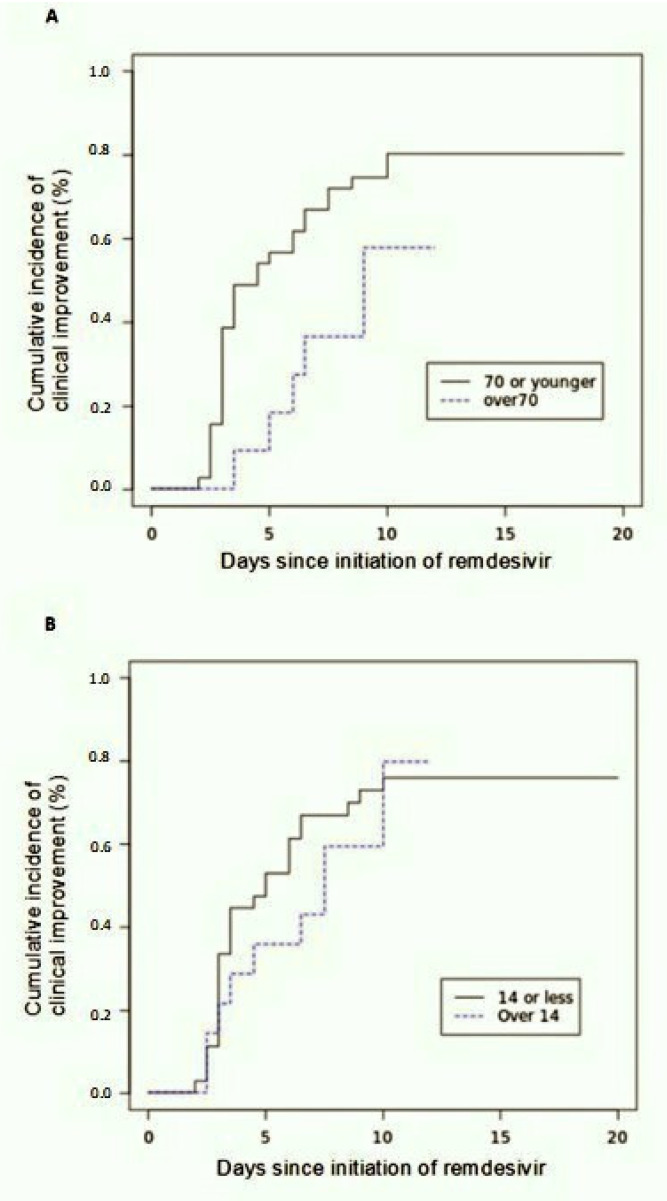
Cumulative incidence of clinical improvement. (**A**) stratified by age. (**B**) stratified by radiologic total severity score at baseline.

The follow up at day 12 showed an improvement in the category of oxygen support in 27 out of 51 patients (53%), whereas 17 patients (33%) still needed CPAP/HFNC and seven subjects (14%) worsened. Among improved subjects, seven were receiving low flow oxygen and 20 were breathing room air. By the date of most recent follow up, 28 of 51 patients (55%) were discharged and seven subjects (13%) died. At day 12 the cumulative incidence of clinical improvement was 43% (95% confidence interval (CI), 24 to 50) by Kaplan–Meier analysis. By 20 days of follow-up, the cumulative incidence of clinical improvement was 71% (95%CI, 55 to 92). Clinical improvement was less frequent among patients older than 70 years (hazard ratio as compared with patients 70 years of age or younger: 0.42; 95% CI, 0.075 to 1.55) (Figure 3A), and among patients with a more severe radiologic score (TSS > 14) (hazard ratio as compared with TSS 14 or less: 0.77; 95% CI, 0.12 to 1.8) (Figure 3B).

A total of 21 patients (41%) reported adverse events during the study. The most common adverse events were increased hepatic enzymes and diarrhea. Renal impairment was observed in one case. Electrolytic abnormalities were observed, such as hyperkalemia (1) and hypernatremia (1). Heart rhythm abnormalities were observed in nine patients (17%), including bradycardia (3), tachycardia (2), atrial fibrillation (1), T waves inversion (1), supraventricular extra systole (1), and ventricular bigeminy (2). A total of six patients (11%) had serious adverse events. Multiple organ dysfunction syndrome, septic shock, cardiogenic shock and hypotension were reported. One subject discontinued Remdesivir treatment prematurely.

## 4. Discussion

COVID-19 symptoms can vary widely. Some people have no symptoms at all, while others become so sick that they eventually need mechanical assistance to breathe, but the majority of patients can experience a progressive change in their symptoms from a less aggressive clinical presentation to a severe clinical form. There is an international consensus about the risk of developing dangerous symptoms of COVID-19 in people who are older and with comorbidities such as heart or lung conditions, weakened immune systems, severe obesity, or diabetes, and this is similar to what is seen with other respiratory illnesses, such as influenza. Furthermore, patients may develop other disorders such as venous thromboembolism that may impair lung performance. So an early identification of subjects which can vary their symptoms and clinical form is important and may depend on anamnestic interview and on viral load. In particular, the viral load is important because it may induce physicians to have a different therapeutic approach: antiviral drugs, in fact, may be associated with typical treatment based on antibiotics, corticosteroids, low molecular weight heparin and oxygen support.

Regarding antiviral therapy, several drugs have been suggested for the treatment of COVID-19, but only Remdesivir has been approved after a positive effect showed in a clinical trial [10]. Remdesivir, in fact, was the first drug approved by the FDA for treating the SARS-CoV-2 virus. It is indicated for treatment of COVID-19 disease in hospitalized adults and children aged 12 years and older who weigh at least 40 kg. Fullapprovalwas preceded by the US FDA issuing an EUA (emergency use authorization) to allow the prescribing of Remdesivir for severe COVID-19.

This compassionate-use study describes clinical outcomes in a small group of severe COVID-19 patients who received NIV and Remdesivir for 5 days. Particularly, a de-escalation of oxygen-support status was observed in 53% of subjects after one week, and the overall mortality was 13% over a follow up of 20 days. These observations are consistent with a recent multicentric study where the 28-day mortality was 13%; in the same study the authors observed a clinical improvement in 84% after a a 10 day course of Remdesivir [11]. In our study clinical effectiveness was 71% at 20 days but treatment was administered for only 5 days, thus supporting the idea that a short course is similarly effective in severe COVID-19 [12].

Beigel et al. reported that clinical improvement among non-invasively ventilated patients after Remdesivir is similar to placebo with a rate ratio of 1.09. The median duration of NIV was 6 days [10,13]. Nevertheless, we observed an overall positive trend of oxygenation, even in more compromised patients. Over a median follow up of 20 days, 66% of patients were breathing room air. On the other hand, a minor rate of clinical improvement was observed in elderly patients (>70 years) and those with a more extensive radiologic involvement of the lungs (TSS > 14). These observations indicate that inclusion criteria may be modified in order to optimize access to cures during this pandemic. Adverse events were reported in 41% during the study. Serious adverse events were reported in 11%, but are likely related to COVID-19 more than to the investigational drug. Moreover, the mortality was 13% and this data seems to be improved if compared to other cohorts from similar geographic areas and similar wards [14]. However, the mortality of patients with severe COVID-19 may also be influenced by the overlapping with venous thromboembolism and pulmonary embolism [15,16,17,18,19,20]. Per our experience, mortality in patients treated with Remdesivir is essentially lower than the 20% observed in the population admitted overall for ARDS to our department in the same period of time. Remarkably, the overall pooled mortality was estimated to be 39% in a recent systemic review of SARS-CoV2 related ARDS [21].

Our study demonstrated that an improvement of severe COVID-19 is possible when Remdesivir is associated with standard treatment. A full clinical improvement, in fact, has been demonstrated by our data regarding clinical presentation and lung performance as far as laboratory markers of acute and sub-acute inflammation (i.e., CRP, IL-6 and so on), and as far as radiological damage.

Of course, the interpretation of the results of this study is limited by the small size cohort but the clinical impact could be considered strong because the population was represented by patients with severe COVID-19 with associated comorbidities. Differences in pharmacological protocols worldwide and ventilatory support should not be considered as biased because these kinds of treatment are used in all patients with COVID-19. Therefore, this study suggests that Remdesivir brings a potential benefit as add-on therapy in severe COVID-19 patients, especially adults with a moderate lung involvement at HRCT. Whether there is a chance for an early treatment with this drug is still matter of discussion and it may depend on several variables such as the viral load, and should be evaluated in further studies.

## 5. Study Limitations

The main study limitation is the absence of a control group; the comparison can only be observed between Remdesivir itself and the adjunctive therapy with immunoglobulin. In addition, there is a limit associated with the small number of observed patients that should be increased in further studies in order to better understand the potential advantages induced by this treatment.

Furthermore, a clinical differentiation is usually made for patients with confirmed COVID-19, regarding severity of lung dysfunction, and should be considered also for the viral load that could be considered before choosing, or not, an associated antiviral treatment. In this way, Remdesivir should be administered to patients in early stage of disease and/or in patients with different viral load in order to be more selective in its functions.

Moreover, as in all real-world studies, the present study may have a number of technical limitations. Data collection is usually performed electronically and sometimes electronic data may be inconsistently collected, with missing data that could be considered relevant at a second time; this may induce a reduced statistical validity and a decreased ability to answer the research question. In real-world trials, in fact, selection bias includes different therapeutic approaches, including off label use of such drugs, tailored therapies prescribed for patients with particular clinical characteristics (e.g., severity of disease and/or other patient characteristics), or any other type of information bias such as misclassification of data or detection bias; in addition, as with all real-life studies, results may be influenced by the absence of a control group.

Further studies are needed to confirm the results reported in this clinical study.

## Figures and Tables

**Table 1:** Baseline epidemiologic and clinical characteristics. CRP: C-reactive protein. IL-6: interleukin-6.TSS: total severity score at HRCT.

Characteristics	Full Cohort
Patients	51
Male (%)	45 (88)
Medianage (IQR)	64 (17)
Mean BMI (IQR)	28 (7)
Co-existing conditions–n.	
0	8
1	16
>1	29
Systemic hypertension	22
Obesity	15
Diabetes Mellitus	12
COPD	8
Arrythmias	2
Cancer	2
PaO2/FiO2	101 (68)
Oxygen support category–n. (%)	
CPAP	40 (78)
HFNC	11 (22)
Median laboratory values (IQR)	
CRP mg/dL	11 (7.6)
IL-6 pg/dL	38.3 (48.5)
Median TSS (IQR)	12 (5)
